# Building the future of biophotonics through experiential education and seasonal schools

**DOI:** 10.1117/1.JBO.31.11.113505

**Published:** 2026-06-18

**Authors:** Patience Mthunzi-Kufa, Jacqueline Maphutha, Zenande Mcotshana, Phumulani Mcoyi

**Affiliations:** a University of South Africa Engineering and Technology, College of Science, School of Science, Florida, South Africa; b Council for Scientific and Industrial Research, Department of Biophotonics, Pretoria, South Africa

**Keywords:** biophotonics, seasonal school, experiential education

## Abstract

**Significance:**

Photonics represents a low-hanging fruit and a highly lucrative industry on the global stage, with applications spanning healthcare and biophotonics, energy and environmental photonics, agriculture and food systems, quantum photonics, telecommunications and data communications, among many other sectors. Despite this breadth of opportunity, the availability of structured biophotonics courses and programs remains limited, largely due to the inherently multidisciplinary nature of the field. Compounding this challenge, many schools, particularly in developing countries, lack adequate science laboratories, especially at the secondary level, thereby depriving learners of the practical exposure necessary to grasp fundamental theoretical concepts meaningfully.

**Aim:**

These gaps underscore the need for innovative and creative approaches to education and capacity development.

**Approach:**

Initiatives such as job-shadowing opportunities, outreach activities, summer schools, and diverse forms of online learning are therefore essential in widening access and building foundational skills.

**Results:**

Introducing photonics, optics, and biophotonics at the high-school and undergraduate levels would significantly strengthen the talent pipeline, cultivating scarce and highly sought-after skills.

**Conclusions:**

Against this backdrop, this paper highlights the importance of targeted strategies that advance biophotonics education and capacity building as a catalyst for sectoral growth.

## 1 Introduction

### 1.1 Growing Need for Cross-Disciplinary Training

**Fig. 1:**
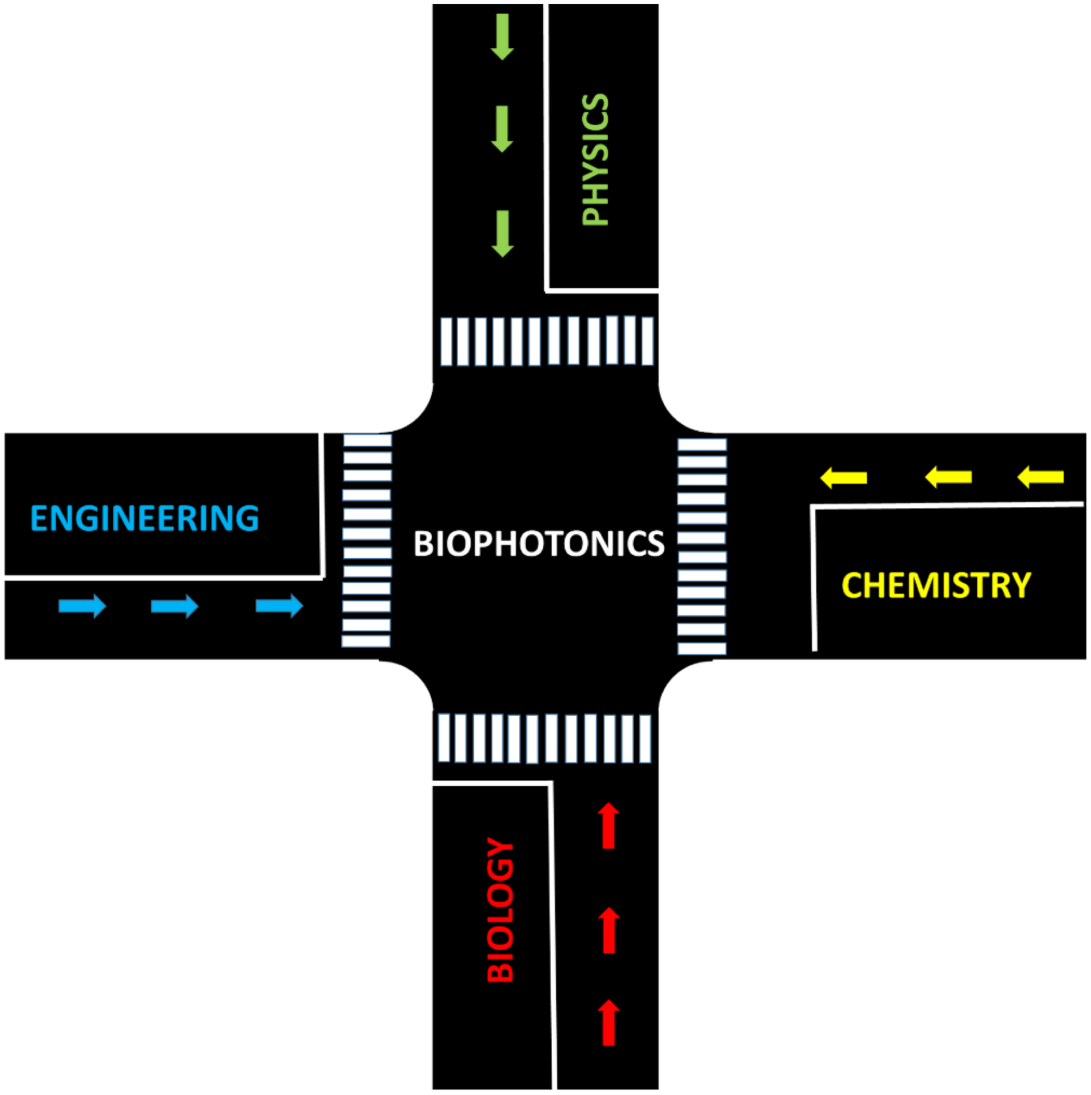
Depiction of biophotonics at the junction of engineering, physics, chemistry, and biology.^2^

Biophotonics is at the forefront of scientific innovation, offering unique insights into biological and biomedical systems. Education serves as a crucial frontier in biophotonics, providing the interdisciplinary expertise necessary to drive scientific discovery and translate photonic technology into biomedical applications.^1^ The growing need for cross-disciplinary training arises from the fact that Biophotonics is a rapidly advancing field that intersects biology, physics, engineering, and chemistry, necessitating cross-disciplinary training. As the field of biophotonics advances, more intricate scientific and technological challenges emerge that cannot be solved by a single discipline. Therefore, researchers must integrate knowledge and expertise from multiple disciplines to acquire problem-solving skills and devise strategies for tackling advanced scientific challenges (Fig. 1).^2^

**Fig. 2:**
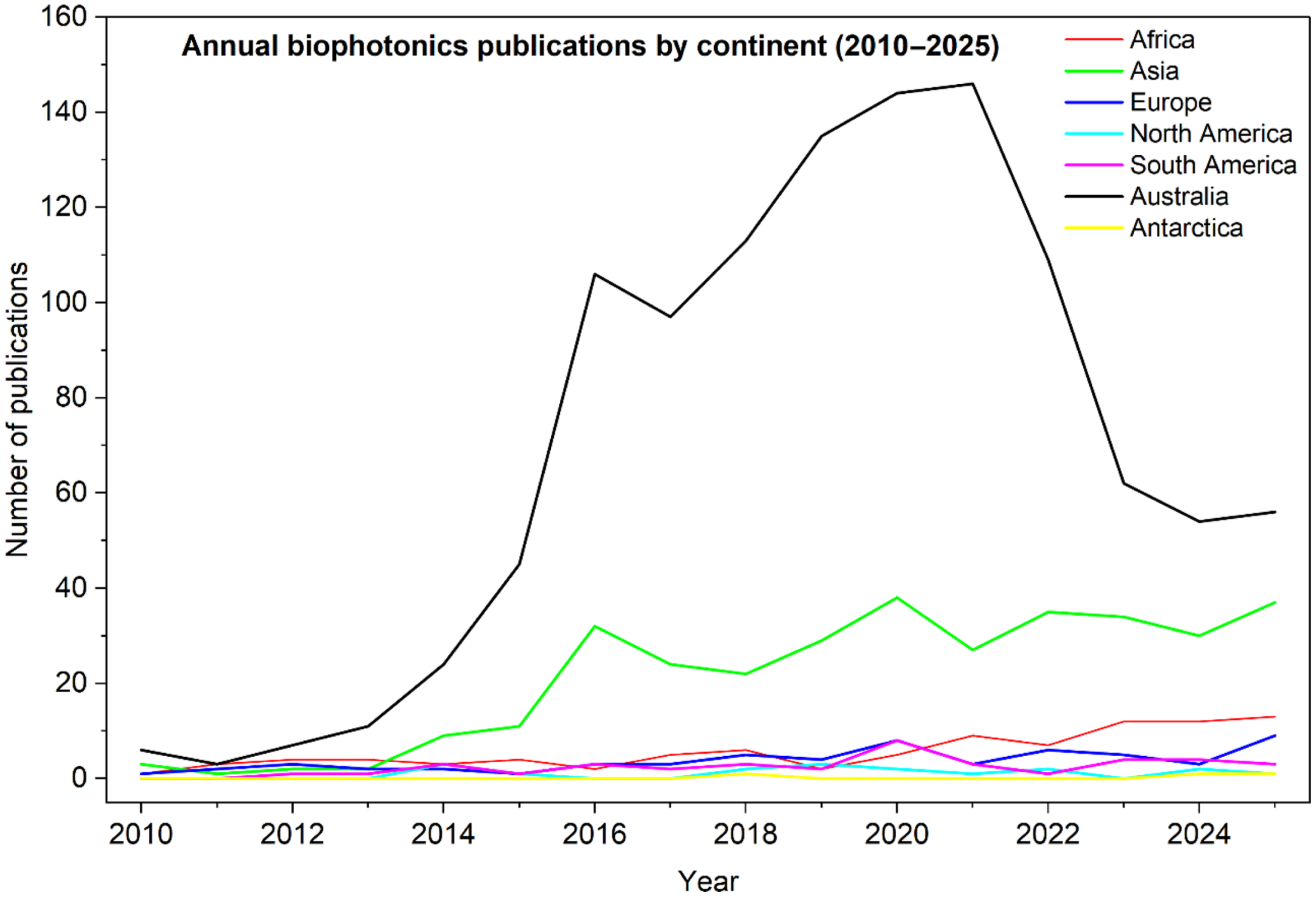
Comparative publication trends in biophotonics across seven continents from 2010 to 2025 (U.S. National Science Foundation, 2023).^3^

Publication trends were obtained from the PubMed database using defined keyword search strategies, and annual publication counts were extracted to compare research output across seven continents (Fig. 2). The analysis reveals a steady global increase in biophotonics publications from 2010 to 2025, with notable regional disparities. Asia demonstrates consistent growth, whereas Australia exhibits a rapid increase followed by a recent decline. Africa shows a gradual upward trend, although overall output remains comparatively low. Europe and North America display relatively stable trends with minor fluctuations, whereas South America shows intermittent increases and Antarctica shows negligible contributions. Overall, these findings highlight the expanding global interest in biophotonics alongside uneven regional development (Fig. 2).

**Fig. 3:**
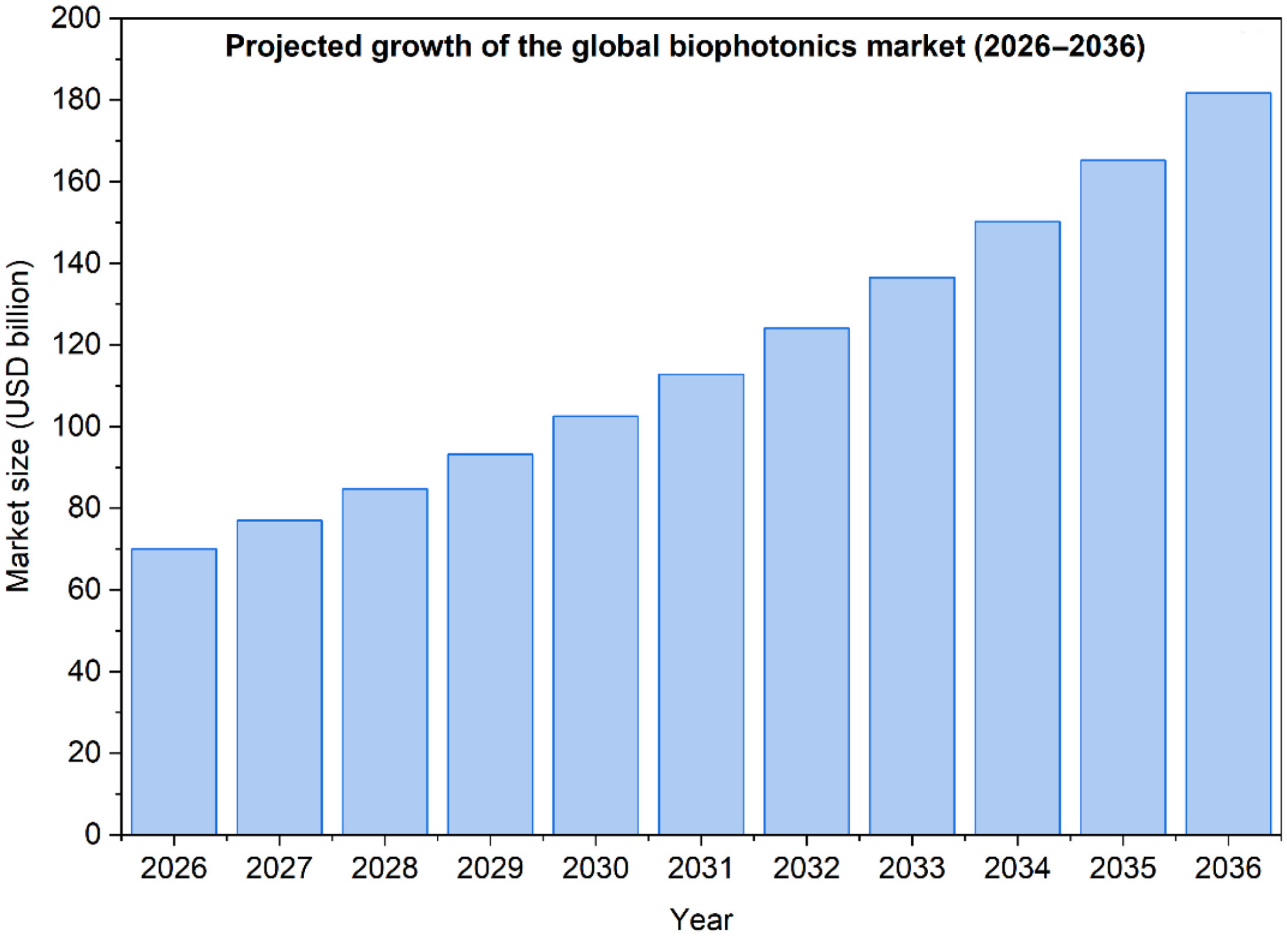
Market projections based on the reported CAGRs from published industry analyses (available online: Ref. 4).

In addition to the observed increase in publication output, the growing importance of biophotonics is further supported by projected market expansion. The global biophotonics market is expected to experience substantial growth over the next decade, increasing from ∼70 billion USD in the mid-2020s to over 100 billion USD by 2030 (Fig. 3). This rapid expansion is driven by increasing demand for advanced diagnostic technologies, non-invasive imaging, and biomedical sensing applications. Such growth underscores the urgent need for interdisciplinary training and expertise in biophotonics to support innovation and meet future technological demands. The projection is based on an assumed compound annual growth rate (CAGR) derived from reported industry estimates.

### 1.2 Gaps in Existing Curricula and the Importance of Hands-On Exposure

Theoretical knowledge and hands-on laboratory experience must be balanced, but a gap still exists between the two, especially at underfunded educational institutions where access to scientific infrastructure and resources is limited. This gap often prevents learners from developing the practical skills needed to operate cutting-edge analytical technologies. Educational institutions and research council organizations can help close this gap by offering specialized programs that cover both theoretical and practical aspects, both offline and online. For example, workshops, educational programs, and professional development courses should be included in these initiatives to provide learners with valuable practical experience and the skills necessary to operate and maintain advanced photonic technologies. In addition, researchers already working in the field should have access to ongoing professional development opportunities that enhance their technical skills and keep them updated on the latest advances in analytical technology.

### 1.3 Purpose and Scope of This Perspective

The purpose of this perspective is to explore how education, rather than being viewed as a supporting component, might act as a strategic catalyst for advancements in biophotonics. Biophotonics occupies a pivotal position between natural and applied sciences, and its advancing development requires a workforce that is both technically proficient and skilled in interdisciplinary logic. The aim is to identify gaps in the current curricula, especially the absence of integrated training and hands-on experience, and integrate experiential learning strategies that blend theory and practical application, encouraging collaboration between organizations such as research councils and academic institutions. Furthermore, academic institutions must ensure that education aligns with the latest developments in technology.

The scope of this discussion includes the following:

• a review of the shortcomings of existing educational approaches in promoting biophotonics
• approaches to improve experiential learning, including industry internships, lab work, and virtual programs
• a call for international collaboration to increase the accessibility and scalability of biophotonics education.

This viewpoint aims to motivate practical actions that will enable the next generation of researchers to enhance biophotonics research and its applications in diverse domains by recognizing education as a critical frontier.

## 2 Role of Seasonal Schools in Advancing Biophotonics

### 2.1 How Seasonal (Summer, Spring, Autumn, and Winter) Schools Bridge Disciplines and Connect Research Communities

Biophotonics, a multidisciplinary field, bridges physics, engineering, life sciences, medicine, and agriculture.^1^ Through seasonal schools, students, early-career researchers, and senior researchers (participants) have an extended time frame to discuss ideas and strategies for realizing these ideas. Furthermore, through various pedagogical approaches, participants in summer schools from different disciplines often engage in tasks that require resolution through interdisciplinary strategies, enabling them to understand the distinct vocabularies, methodologies, and analytical techniques of different disciplines. Seasonal schools also enhance accessibility to state-of-the-art biophotonics technologies, especially for participants from low- and middle-income countries (LMICs).^5^ Seasonal schools, such as the Society of Photo-Optical Instrumentation Engineers (SPIE) Biophotonics Summer School, biennially held in Sweden, offer keynote lectures from leading researchers, expert lectures, and hands-on workshops to foster the next generation of biophotonics researchers.^6^^,^^7^ In addition to the SPIE Biophotonics Summer School, the Siegman International School on Lasers,^8^ Biocube Workshop and Winter School,^9^ and Max Planck School of Photonics Autumn School^10^ are seasonal schools that also foster the next generation of biophotonics researchers. International seasonal schools are crucial for participants, as they fill research gaps and foster collaborations, but most seasonal schools are distributed in Europe, making it inaccessible to some researchers from LMICs.^5^ The lectures at seasonal schools set the knowledge benchmark for participants, standardizing terms and concepts globally. Biophotonics technologies are evolving rapidly, and seasonal schools provide participants with the opportunity to stay current with the latest innovations. Essentially, seasonal schools serve as incubators for interdisciplinary partnerships, which are fundamental to the field of biophotonics. The distribution of seasonal schools is high in Europe but various seasonal schools also exist in LMICs, thereby developing biophotonics beyond Europe.

### 2.2 Pedagogical Approaches: Immersive, Interdisciplinary, and Project-Based Learning

Three pedagogical approaches (immersive, interdisciplinary, and project-based learning) are crucial for seasonal schools. They enhance collaboration, deepen concept understanding, foster creativity, promote research independence, and establish new networks. Immersive learning provides hands-on access to state-of-the-art biophotonics instrumentation, live demonstrations by experts, and real-world experimental scenarios, fostering active learning and confidence in navigating complex experimental challenges.^11^ Interdisciplinary learning fosters innovative thinking and problem-solving by understanding broad interdisciplinary connections among medicine, biology, physics, and engineering.^12^ Biophotonics, a multidisciplinary field, benefits from interdisciplinary learning. Seasonal schools, such as the SPIE Biophotonics Summer School, aid in this learning. Project-based learning teaches students and researchers how to work in teams and design projects with clear problem statements, deliverables, and timelines.^13^ These fundamental pedagogical approaches equip both students and researchers with the capabilities to navigate complex biophotonics challenges with confidence, creativity, and real-world competence.

### 2.3 Examples of Impactful Programs and Outcomes

The SPIE Biophotonics Summer School, an impactful international biennial program, selects emerging researchers from various disciplines (optics, engineering, and life sciences).^7^^,^^14^ Alumni report rapid upskilling in biophotonics instrumentation and interdisciplinary problem-solving. The program also supports early-career researchers in collaborations and enhances their capabilities in utilizing advanced biophotonics technologies.^7^^,^^14^ The St. Andrews Biophotonics Summer School offers hands-on training in various biophotonics technologies.^15^ Alumni benefit from seasonal schools include upskilling, enhanced understanding of methodologies, increased networking capacity, prospects for co-supervision, and research visits.^7^ The BIGGS Biophotonics and Imaging Graduate Summer School offers expert lectures and highly interactive poster sessions.^16^ The East African Summer School on Optics and Lasers organized by the International Centre for Theoretical Physics (ICTP) sought to increase the number of individuals with lasers and photonics research expertise to help further develop photonics research on the African continent. ICTP serves as an example to highlight how seasonal schools play a pivotal role in the development of biophotonics research in LMICs.^17^ The ICFO-KNUST International School on the Frontiers of Light in Kwame Nkrumah University of Science Technology (KNUST), Kumasi, Ghana, offers lectures, discussion sessions, and professional development workshops, further highlighting some seasonal schools available in LMICs.^18^

## 3 Emerging Educational Innovations and Global Perspectives

### 3.1 Post-Pandemic Hybrid Models and Virtual Training

Global education is undergoing seismic change. Traditional models of schooling are rapidly losing relevance.^19^ Fresh insights into how people learn demand bold innovation in teacher preparation, school systems, and beyond. Worldwide, educators are responding to complex cultural, political, and socio-economic challenges with new ideas and practices. These innovations are as diverse as the contexts from which they emerge, distinct in every community yet connected across borders. Some draw on shared principles such as transformation, collaboration, and coherence; others grow from long-standing educational philosophies reimagined for a new era.^20^

Real, system-wide reform begins with a blunt truth: the system itself must change. The post-pandemic world has made that impossible to deny. In 2020, Coronavirus disease-2019 (COVID-19) disrupted the global education status quo. Almost overnight, schools and universities shut their doors.^21^ The familiar rhythm of classrooms and face-to-face teaching vanished, forcing the world to confront fundamental questions: What is education? How should it happen? Who is it really for? What followed—widely labeled “emergency remote teaching”—was never a carefully planned transition to online learning. It was crisis management. In the chaos, the cracks in the system were laid bare: educators unprepared for digital instruction, deep digital divides both between countries and within individual schools, and a fragile infrastructure not built for disruption. Today, education is slowly emerging from the shock of COVID-19. However, this is no time to “go back to normal.” The sector stands at a crossroads: either slide back into old patterns or seize this moment to hardwire the lessons learned into a stronger, more equitable, and truly pandemic-ready model for the future.^22^

The explosive rise of remote learning exposed both the enormous power of digital tools and the hard limits of how they are used in education. It also forced schools to adopt technology on a scale that had seemed impossible just a few years earlier. Platforms such as Microsoft Teams, Moodle, and Zoom quickly moved from optional extras to the backbone of emergency learning systems. That period demonstrated that technology could sustain education under extreme pressure and provide teachers and students with new forms of flexibility. At the same time, it laid bare the digital divide in its starkest form.^23^ The digital divide refers to the gap between individuals who have access to modern technology and those who do not. To truly participate in learning, students require more than just content; they need reliable internet, a suitable learning environment, and a digital device.^24^ The emergency phase made it obvious that simply uploading lessons to a content library is nowhere near enough. Students and teachers need a full ecosystem of support, connectivity, and well-designed digital experiences. Integrating technology into hybrid learning is not just a technical upgrade; it demands a fundamental shift in pedagogy. Traditional, lecture-heavy methods often fail to meet the diverse needs of learners. Instead, classrooms must be equipped with multiple learning models tailored to how students learn.^23^^,^^24^

### 3.2 Integration of Artificial Intelligence (AI), Computational Optics, and Data Literacy in Teaching

Here, Vygotsky’s constructivist theory offers a powerful foundation. In hybrid environments, instructors design activities that promote collaboration, problem-solving, and hands-on exploration. Their role shifts from being the main source of knowledge to becoming facilitators and coaches, especially in helping students master digital tools.^25^ Today, artificial intelligence, computational optics (including programmable lighting, virtual reality (VR) optics, and simulations), and data literacy are converging to transform how we teach across disciplines. Focused integration of these three domains has helped to radically modernize education in the post-pandemic era, as schools have been forced to adapt continuously.^26^ The post-pandemic classroom is no longer confined by walls. Its limits are defined not by physical space but by the reach, design, and inclusivity of digital education.

### 3.3 Lessons from Diverse Regions: Sustainability, Inclusivity, and Scalability

In highly diverse regions such as India, bold post-pandemic initiatives are reshaping education. The COVID-19 pandemic has accelerated the deployment of digital education and has encouraged the Indian government to increase access to learning content in different levels of connectivity, including online, limited-access and offline environments. Platforms such as DIKSHA, SWAYAM, and E-Pathshala remain pivotal initiatives as they provide free digital content in different languages to millions of learners in urban and rural schools. Despite these developments, the digital divide remains a defining challenge, with only 34% of rural households having internet access as of 2023, indicating that scaling digital literacy requires not just technology deployment but sustained investment in infrastructure and equity.^27^ Elsewhere, countries such as Finland and Estonia are investing heavily in the future with national AI-in-education strategies. From grade 7 onward, every learner is now required to study engineering and data literacy as core subjects, ensuring entire generations grow up fluent in the language of technology.^28^ England, Czechia, Korea, Spain, and Turkey are providing both digital material and virtual classes post-pandemic, which were initially developed during the pandemic, and their success has carried over beyond the pandemic. As such, Spain has developed a Digital Spain 2025 project for continuous digitization and virtual classes. This project has resulted in the development of an online website (Aprendo en casa), which provides digital materials to students from primary, secondary, and vocational education and training (VET) levels and offers guidance to teachers as well. This website is further supported by the International Equal Education Foundation, which also supports teachers with digital materials. To scale up these initiatives, the Spanish Ministry of Education and VET is collaborating with regional governments to connect more schools and improve access.

### 3.4 Statistical Evidence of the Digital Shift

The global pivot toward remote and digital education post-COVID is well documented in the literature. For instance, UNESCO reported that in 2020, over 1.6 billion learners in more than 190 countries were affected by school closures, with digital platforms becoming the primary mode of instruction for nearly 70% of higher education institutions worldwide.^29^ In the United States, surveys indicated that 73% of universities adopted hybrid or fully online models during the pandemic, compared with <20% prior to 2019.^22^^,^^23^ Similarly, in Europe, the European Commission noted that over 85% of students engaged in some form of online learning during the pandemic, with many institutions continuing to offer virtual classes beyond 2022.^30^ These statistics illustrate both the scale and permanence of the digital shift, reinforcing the argument that although digital tools democratize access, they must be complemented by hands-on experiential learning to ensure comprehensive training in biophotonics.

### 3.5 Balancing Digital Innovation with Hands-On Experience

Although digital and hybrid models have proven transformative in widening access to education, particularly during and after the COVID-19 pandemic, it is important to acknowledge their limitations in experimental fields such as biophotonics. Theoretical knowledge acquired through online platforms cannot fully substitute for the tactile, practical engagement required to operate advanced photonic instrumentation. Many universities and research institutes worldwide recognized this reality and, in the aftermath of the pandemic, deliberately reduced or prohibited extended “home–office” arrangements for students and scientists in experimental disciplines. Their rationale was clear: without direct laboratory exposure, learners risk developing only partial expertise, which constrains both their technical competence and their ability to innovate. Addressing this risk underscores the need for hybrid models that integrate digital flexibility with structured, in-person laboratory training. This perspective links directly to the funding and infrastructure challenges discussed in Sec. 4.1, highlighting that equitable access must encompass not only digital connectivity but also physical opportunities for hands-on learning.

## 4 Challenges and Opportunities

### 4.1 Funding and Infrastructure Limitations

Despite the increasing demand in biophotonics, institutions still bear substantial limitations, including instrumentation, technical support, and limited laboratory space. Biophotonics relies on optical benches, lasers, spectrometers, and computational systems, thus requiring funding support far beyond what most institutions can afford. Summer schools, which normally offer structured and condensed hands-on teaching, suffer the same fate of constrained funding. Furthermore, the lack of infrastructure for research for centralized biophotonics centers limits the potential of experiential learning. Thus, addressing these issues requires coordinated government interventions, particularly from the Ministries of Finance and Education, to establish long-term funding models.

### 4.2 Ensuring Equitable Access and Regional Capacity Building

Equitable access and regional capacity in experiential education and seasonal schools can be achieved by providing funding (travel grants and scholarships) for applicants, especially applicants from LMICs.^31^^,^^32^ Sourcing equipment locally and training local facilitators to reduce recruitment costs in the context of seasonal schools or experiential education (e.g., research visits). Implement standard operating protocols and extensive lab protocols (with more detail and troubleshooting tips) for hands-on training and research visits. If grants are limited, record and share or live-stream content to ensure equitable access. Consider the language barrier for international seasonal schools and research visits and provide translations or subtitles. Establish regional hubs for researchers and students to further expand their expertise in biophotonics following seasonal schools or research visits. Establish an alumni network to track upskilling and publications and use this to secure more funding for equitable access strategies and regional hub development.

### 4.3 Collaboration with Professional Societies, Industry, and Policy Stakeholders

The challenges that result from collaborating with professional societies, industry, and policy stakeholders include a lack of coordination, which results in redundant efforts and lost opportunities. Academic programs often fail to integrate real-world case studies or internships that align with industry requirements, resulting in a skills gap. Collaboration with these stakeholders can facilitate knowledge exchange, resource sharing, and collaborative problem-solving. Conference attendance and participation in professional networks can help researchers and developers stay up to date on the newest developments and establish valuable partnerships.

## 5 Future Outlook: Toward a Global Biophotonics Learning Ecosystem

### 5.1 Vision for Connected Educational Networks and Shared Open Resources

The future of biophotonics education lies in the development of a truly global learning ecosystem that is collaborative, resource-efficient, and inclusive. Drawing inspiration from the traditional library system, where knowledge, infrastructure, and stewardship are shared rather than duplicated. Shared biophotonics facilities can serve as distributed “knowledge hubs,” enabling institutions to access advanced instrumentation, datasets, and training platforms without prohibitive capital investment. Through coordinated collaboration frameworks, universities, research centers, and industry partners can pool specialized equipment and expertise. Meanwhile, structured student and staff exchange programs allow learners to rotate across nodes of excellence, gaining hands-on experience with diverse technologies and applications. Such mobility not only maximizes the use of existing human capital and infrastructure but also accelerates the transfer of skills between students and experts in the field, standardizes training, and fosters the co-creation of knowledge across borders. Ultimately, a globally networked biophotonics learning ecosystem will democratize access to high-end photonic education, strengthen capacity in resource-limited regions, and cultivate a new generation of scientists equipped to address pressing health and societal challenges.

### 5.2 Strategic Recommendations for the Next Decade

Over the next decade, the sustainable growth of biophotonics will depend on the deliberate expansion of experimental learning pathways and well-coordinated summer schools that function as incubators for skills, innovation, and leadership. Integrated strategies should be co-designed by key stakeholders, including universities, research institutes, industry partners, funding agencies, professional societies, and government, to align training with emerging scientific and societal needs. Experiential programs must prioritize hands-on laboratory exposure, problem-driven learning, and cross-disciplinary collaboration. Meanwhile, summer school alumni networks can play a transformative role by “paying it forward” through mentorship, fundraising, and resource mobilization for future cohorts. To ensure global equity, dedicated mechanisms should support participation from the developing world, including travel grants, shared facilities, hybrid delivery models, and regional hubs that leverage existing infrastructure. Embedding principles of transformation, inclusivity, and diversity across all programs through targeted outreach, equitable selection processes, and sustained support will be essential in building a resilient, globally connected biophotonics workforce capable of advancing health, innovation, and socio-economic development.

## 6 Conclusion

This paper underscores biophotonics as both a strategic scientific frontier and a powerful catalyst for socio-economic and health-related impact while highlighting persistent gaps in education, infrastructure, and equitable access to training. The analysis demonstrates that experimental learning, summer schools, outreach initiatives, and early exposure at secondary and undergraduate levels are essential in addressing the multidisciplinary barriers that currently limit participation and skills development. By adopting collaborative, library-inspired models of shared facilities, fostering global and regional partnerships, and mobilizing alumni-driven sustainability mechanisms, a resilient and inclusive biophotonics learning ecosystem can be realized. Importantly, prioritizing transformation, diversity, and support for the developing world is not only a moral imperative but also a strategic investment in the future workforce. Collectively, these insights underscore the need for coordinated, long-term strategies that integrate education, research, and community engagement to ensure the continued growth and global relevance of biophotonics over the coming decade.

## Data Availability

Data sharing is not applicable.
